# Zoonotic Bacterial Respiratory Infections Associated With Cats and Dogs: A Case Series and Literature Review

**DOI:** 10.7759/cureus.24414

**Published:** 2022-04-23

**Authors:** Lauren E Rybolt, Suhel Sabunwala, John N Greene

**Affiliations:** 1 Internal Medicine, University of South Florida, Tampa, USA; 2 Infectious Disease, University of South Florida, Tampa, USA; 3 Infectious Disease, Moffitt Cancer Center, Tampa, USA

**Keywords:** zoonotic respiratory infections, zoonotic infections, zoonotic pneumonia, zoonotic bacterial pneumonia, zoonotic diseases in cats, zoonotic diseases in dogs, immunocompromised patients, pasteurella multocida, bordetella bronchiseptica

## Abstract

Cats and dogs make up an essential part of the household for families in the United States. Close contact with pets can carry a risk of potential infectious disease transmission. This case series outlines causes of zoonotic pneumonia associated with cats and dogs, with a particular focus on the three cases presented of respiratory infection with *Bordetella (B.) bronchiseptica* and *Pasteurella (P.) multocida* in patients with an underlying malignancy. *B. bronchiseptica* is a rare bacterial pathogen in humans that can cause disease in immunocompromised individuals. Interpreting the significance of *B. bronchiseptica* as a pathogenic agent can be challenging given that this microbe often accompanies other organisms in culture. *P. multocida* is another important pathogen known to cause severe respiratory infection in immunocompromised populations or those with certain underlying comorbidities.

A broadened differential for other bacterial etiologies of zoonotic respiratory infection acquired from dogs or cats includes* Francisella tularensis, Yersinia pestis, Coxiella burnetii,* and *Bartonella henselae*. These pathogens should be considered in the correct clinical context. Pets also play a role as reservoirs for the transmission of resistant bacteria such as methicillin-resistant *Staphylococcus aureus* (MRSA), methicillin-resistant *Staphylococcus intermedius* group (SIG), and extended-spectrum β-lactamase (ESBL)-producing Enterobacteriaceae. Immunocompromised individuals must be educated on the potential for household transmission of zoonotic disease and how to limit certain types of close contact with pets. This report also highlights the importance of flea and tick control in pets for the prevention of zoonotic disease spread.

## Introduction

Dogs and cats are some of the most common household pets in the United States. While pets are known to provide cheer and companionship to many homes, their potential for infectious disease transmission is also well-known. Much of the current literature focuses on bite-and-scratch wound-related skin and soft tissue infections. However, recognition should additionally be given to respiratory infections acquired from pets. Special consideration of zoonotic bacterial pneumonia is of particular importance in susceptible immunocompromised, elderly, and critically ill patients. Furthermore, cats and dogs are increasingly being acknowledged as potential reservoirs for the transmission of resistant bacteria, particularly methicillin-resistant *Staphylococcus aureus* (MRSA), methicillin-resistant *Staphylococcus intermedius* group (SIG), and extended-spectrum β-lactamase (ESBL)-producing Enterobacteriaceae, which all have the ability to cause pulmonary disease [1-3]. Here, we examine bacterial etiologies of pneumonia acquired from cats and dogs with a focus on three cases presented with respiratory infection caused by *B. bronchiseptica *and *P. multocida* in patients with an underlying malignancy.

## Case presentation

Case one

A 65-year-old woman with a history of multiple myeloma refractory to various therapy modalities initially presented to an outside hospital after being found down for an unknown duration with significant oral and rectal bleeding. She had a previous history of autologous hematopoietic stem cell transplantation (HSCT) followed by disease progression two years later and was currently on therapy with belantamab mafodotin. On arrival, she was severely anemic and thrombocytopenic. After stabilizing with blood transfusions, endoscopy revealed an esophageal mass and ulcers without active bleeding. Biopsies of the mass were not taken given the precarious risk of uncontrolled bleeding. The patient was also found to be bacteremic with blood cultures positive for methicillin-sensitive *Staphylococcus aureus *(MSSA) and *Clostridium bifermentans*. She was noted to have an allergy to penicillin that included severe skin peeling, as well as allergies to sulfa drugs and levofloxacin, therefore an antibiotic regimen of IV vancomycin, aztreonam, and metronidazole was chosen. The patient was then transferred to a cancer specialty hospital for further management.

**Figure 1:**
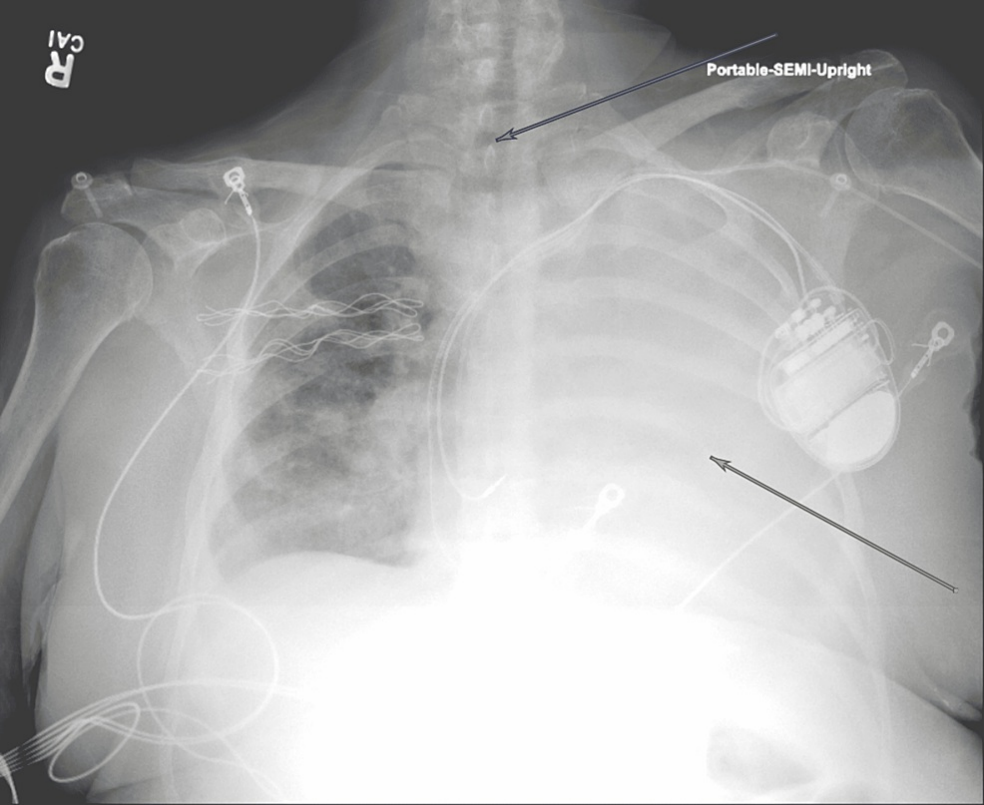
Initial chest X-ray upon hospital transfer demonstrating complete opacification of the left thorax (yellow arrow) with slight right-sided mediastinal shift (blue arrow)

Upon transfer, she was tachycardic, mildly tachypneic, afebrile, and normotensive, with an oxygen saturation of 100% on 15L OxyMask^TM ^(Southmedic Inc, Canada). The patient appeared drowsy, with waxing and waning consciousness. Crackles were present in the right lower lung field with minimal breath sounds auscultated in the left chest. Laboratory results on arrival were notable for hemoglobin 8.6 g/dL, platelets 23 X 10^9^/L, international normalized ratio (INR) 2.5, and normal white blood cell (WBC) count of 5.75 X 10^9^/L. Initial imaging with chest X-ray (Figure 1) demonstrated complete opacification of the left thorax with a mild right-sided mediastinal shift. Shortly after arrival, the patient required intubation due to increasing oxygen requirements and an inability to protect their airway.

**Figure 2:**
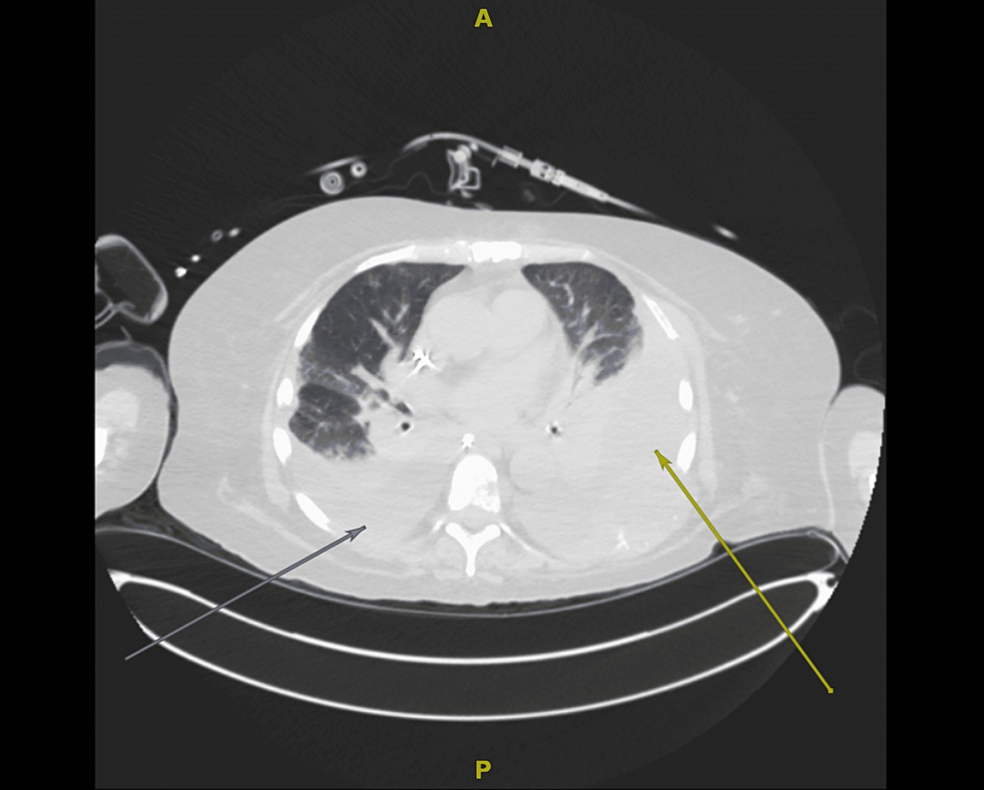
Axial section from a non-contrast CT chest showing bilateral pleural effusions (arrows) and ground-glass opacities

After intubation, the initiation of two vasopressors was required. On day two after the transfer, the patient was started on continuous renal replacement therapy for fluid overload with a poor diuretic response. A non-contrast CT of the chest (Figure 2) was obtained, showing a large left pleural effusion with left lower lobe collapse, a small-moderate right pleural effusion with partial atelectasis, and ground-glass opacities involving bilateral lung fields. A thoracentesis was considered; however, this was not pursued in the setting of risks with the patient being coagulopathic and thrombocytopenic. The effusions were suspected to be malignant in etiology. The ventilator settings were able to be weaned to a minimum. Despite this improvement, the patient was precluded from extubation due to severe encephalopathy that was demonstrated on electroencephalography (EEG). CT head imaging as part of the workup for encephalopathy was unrevealing.

The antibiotic regimen from the outside hospital was initially continued but later de-escalated to IV vancomycin monotherapy. Repeat blood cultures were negative for growth, but a tracheal aspirate culture obtained at admission produced moderate growth (3+) of MSSA. A bronchoscopy was performed 10 days after transfer due to an acute increase in respiratory rate and hypercapnia without any change in ventilator settings. Diffuse, thick secretions were noted in bilateral lung fields with small, punctate hemorrhages in the bronchial aspiration. Bronchoalveolar lavage (BAL) cultures grew 3+ *Bordetella bronchiseptica*, 4+ MSSA, and a few colonies of multidrug-resistant *Klebsiella pneumoniae* with susceptibilities as listed in Table 1. Antibiotics were not adjusted for these findings, as this was thought to represent colonization rather than a true airway infection since her respiratory status remained relatively stable on minimal ventilator settings. Information on possible pet contacts as a source for acquiring *B. bronchiseptica* was unable to be obtained. Ultimately, ongoing discussions with the patient’s healthcare surrogate led to the decision to transition to comfort measures with the withdrawal of life-sustaining care given her overall guarded prognosis.

**Table 1:** Causes of bacterial pneumonia potentially transmissible by cats and dogs * Minimum inhibitory concentration (MIC) via microdilution method. ** Susceptibility interpretation (R-resistant, S-susceptible, I-intermediate)

	Antibiotic	MIC*	Interpretation**
Klebsiella pneumoniae	Ampicillin	>=32	R
	Ampicillin/Sulbactam	>=32	R
	Cefepime	2	S
	Cefoxitin	>=64	R
	Ceftriaxone	>=64	R
	Gentamicin	>16=	R
	Meropenem	<=0.25	S
	Piperacillin/Tazobactam	>=128	R
	Tobramycin	>=16	R
	Trimethoprim/Sulfa	>=320	R
	Ciprofloxacin	>=4	R
Bordetella bronchiseptica	Cefepime	32	R
	Ceftriaxone	>=64	R
	Gentamicin	2	S
	Piperacillin/Tazobactam	64	I
	Tobramycin	2	S
	Trimethoprim/Sulfa	80	R
	Ciprofloxacin	1	S
Staphylococcus aureus	Penicillin	0.25	R
	Ciprofloxacin	<=0.5	S
	Clindamycin	<=0.25	S
	Erythromycin		R
	Gentamicin	<=0.5	S
	Linezolid	2	S
	Oxacillin	<=0.25	S
	Quinupristin/Dalfopristin	<=0.25	S
	Rifampin	<=0.5	S
	Tetracycline	>=16	R
	Tigecycline	0.25	S
	Trimethoprim/Sulfa	<=10	S
	Vancomycin	<=0.5	S

Case two

**Figure 3:**
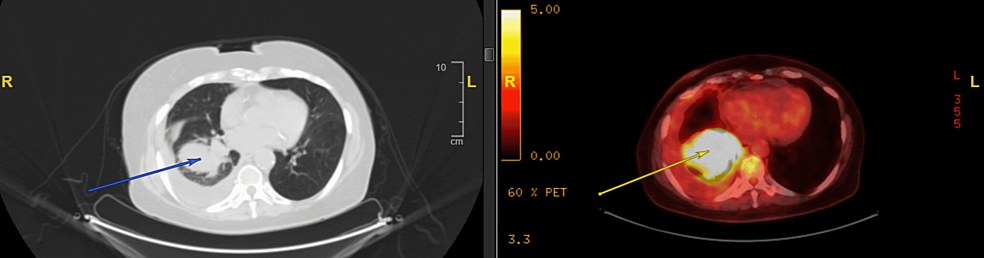
PET-CT scan axial section demonstrating a metabolically active right lower lobe heterogeneous mass (arrows) and right pleural effusion PET: positron emission tomography

**Figure 4:**
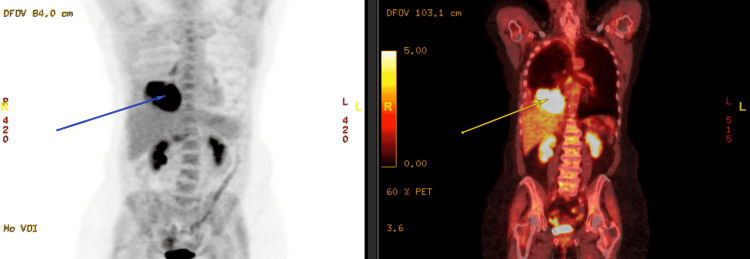
PET-CT scan coronal section demonstrating a metabolically active right lower lobe heterogeneous mass (arrows) PET: positron emission tomography

A 73-year-old woman with a history of chronic obstructive pulmonary disease (COPD) and tobacco use presented to the pulmonology clinic for ongoing workup of a right lung mass that was incidentally noted on low-dose CT chest screening for lung cancer. Initial CT-guided biopsy of the right lower lobe mass was negative for malignancy. The patient subsequently had recurrent right-sided pleural effusions with symptoms of increased shortness of breath requiring thoracentesis on three occasions. The pleural fluid samples were serosanguinous in quality with cytology negative for malignancy and cultures negative for infectious etiology. She had a positron emission tomography (PET)-CT scan (Figures 3-4) demonstrating a metabolically active heterogeneous mass encompassing much of the right lower lobe with obstruction of the right lower bronchus. The PET-CT scan also showed a moderate right-sided pleural effusion, thoracic adenopathy, a metabolically active area at the ninth thoracic vertebra, and irregular non-avid opacities in the perihilar left lower lobe.

Later, the patient underwent outpatient bronchoscopy with an endobronchial ultrasound-guided fine-needle aspiration of lymph nodes. A vascular endobronchial lesion was noted in the left lower lobe, for which a biopsy sample was collected. Right lower lobe BAL washings were also sent. Pathology of the endobronchial lesion, BAL washings, and one of the sampled lymph nodes was consistent with squamous cell carcinoma. In addition, BAL cultures grew 4+ *P. multocida* susceptible to azithromycin, ampicillin, levofloxacin, penicillin, trimethoprim-sulfamethoxazole, and tetracycline. Pet contact was confirmed, as the patient reported she had a dog living at home, but she could not recall any specific incidence of being scratched or bitten. The patient was successfully treated outpatient with a seven-day course of oral amoxicillin-clavulanate.

Case three

A 74-year-old woman with a history of aplastic anemia, prior tobacco use, and chronic lymphocytic leukemia (CLL) presented to the hospital after being found tachypneic and febrile during her outpatient hematology appointment. She had previously been enrolled in a clinical trial for CLL but elected to withdraw due to adverse effects and had been on observation for her illness. Upon arrival, the patient was noted to be disoriented with rigors and fever up to 103°F. The physical exam was notable for diminished bibasilar breath sounds. She had reportedly been in her usual state of health the previous day. However, she had recently been treated for right forearm cellulitis at another hospital after being bitten by a friend’s cat a few weeks beforehand. The patient also had one cat living at home. Fevers and altered mentation had similarly developed during the prior hospitalization. Broad-spectrum antibiotics were initially begun but subsequently de-escalated to a 10-day course of amoxicillin-clavulanate acid. While on antibiotics, there was near resolution of the cellulitis and fevers. Upon completion of the antibiotics, the fevers returned.

Upon admission, her laboratory studies were significant for a WBC count of 13.14 X 10^9^/L with a low absolute neutrophil count (ANC) of 0.79 X 10^9^/L, hemoglobin 7.5 g/dL, platelets 11 X 10^9^/L, and procalcitonin of 2.8 ng/ml. The initial chest X-ray showed no acute cardiopulmonary process. Blood cultures were drawn, and she was started on IV piperacillin-tazobactam. After blood cultures grew *P. multocida*, antibiotics were changed to IV ampicillin-sulbactam and oral doxycycline for double coverage of *P. multocida* pending susceptibility studies.

**Figure 5:**
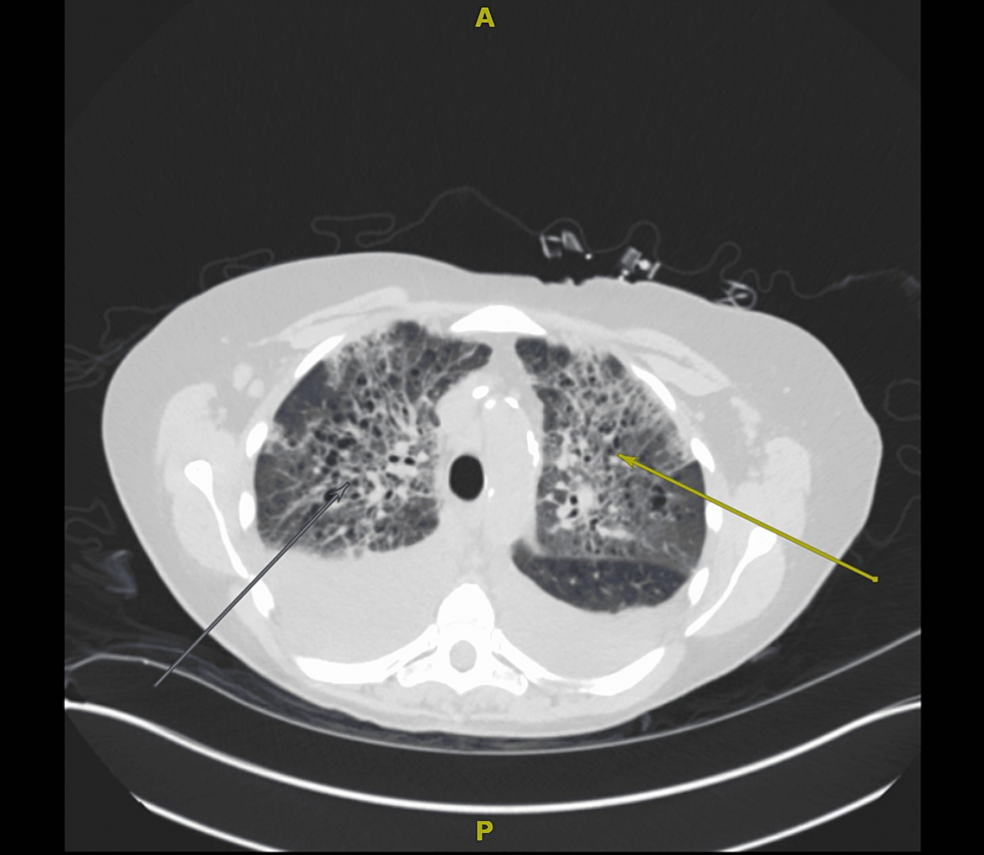
Axial section from a non-contrast CT chest showing large bilateral pleural effusions, interlobular septal thickening, as well as ground-glass and reticular opacities (arrows)

A few days into her hospitalization, the patient began to have new oxygen requirements. A repeat chest X-ray showed new bilateral perihilar interstitial lung markings suggestive of pulmonary edema. An echocardiogram was obtained, revealing an ejection fraction of 35-40%. Notably, the patient had no prior history of heart failure. A CT chest without contrast was also performed, which showed large bilateral pleural effusions, mediastinal plus axillary lymphadenopathy, as well as reticular and ground-glass opacities concerning for an inflammatory process (Figure 5). During this time, the patient experienced some mild hemoptysis and was therefore started on a high-dose steroid taper due to concern for possible diffuse alveolar hemorrhage (DAH). She subsequently underwent a right-sided thoracentesis, which drained 560 ml of dark orange-colored pleural fluid. Pleural fluid cultures remained negative. In addition, a bronchoscopy was performed due to the concern for DAH, which showed only scant old blood and bronchiectasis with mucus plugging. BAL cultures grew 1+ *Candida albicans*, thought to represent colonization.

The patient’s respiratory status improved after the thoracentesis and with continued diuresis. Blood culture susceptibility studies showed that *P. multocida* was sensitive to ampicillin, levofloxacin, and penicillin. Repeat blood cultures remained negative for any growth. Her antibiotics were de-escalated to oral levofloxacin per infectious disease with instructions to continue for a one-month course upon discharge home. The bacteremia was thought to result from chronic pulmonary colonization with *P. multocida*.

## Discussion

Bacterial etiologies of zoonotic respiratory infection that can be acquired from cats or dogs include *Bordetella bronchiseptica, Pasteurella multocida, Francisella tularensis, Yersinia pestis, Coxiella burnetii*, and *Bartonella henselae*. Although these are not common microbes to encounter in a patient presenting with pneumonia, it is essential to consider them in the right context. When considering a differential for pulmonary infection in immunocompromised patients or those with underlying comorbidities, the spectrum of diseases should be broad enough to include these various zoonoses. The presented cases of *B. bronchiseptica* pneumonia in a patient with multiple myeloma as well as *P. multocida* infection in a patient with squamous cell lung carcinoma and a patient with CLL add to the current literature on zoonotic respiratory infections. This discussion also examines some of the other bacterial agents of zoonotic pneumonia potentially transmissible by cats and dogs. Table 2 highlights these agents.

**Table 2:** Overview of bacterial pneumonia etiologies potentially transmissible by cats and dogs *Cats > Dogs indicates that in comparison, cats serve more frequently as a reservoir for infection

Pathogen(s)	Pet Reservoir	Transmission	Notable Features
Bordetella bronchiseptica	Dogs, Cats	Contact with animal [4-7], nosocomial [5]	Increased risk in patients with chronic pulmonary disease or immunocompromising conditions [4-7]
Pasteurella multocida	Dogs, Cats	Contact with animal (scratch, bite/lick, inhalation) [8-10]	Increased risk in elderly, immunocompromised, and patients with a chronic pulmonary disease[9-10]. Pneumonia may be a primary event or secondary to bacteremic spread [8]
Francisella tularensis	Cats > Dogs*	Pet importation of infected ticks [11], contact with an animal (bite, scratch, inhalation) [11-12]	Pneumonia may be a primary event or secondary from bacteremic spread [12]
Yersinia pestis	Cats > Dogs*	Pet importation of infected fleas [13], contact with an animal (bite, scratch, inhalation) [12-13]	Pneumonia may be a primary event or secondary to bacteremic spread [12-13]. Unlike dogs, cats are more likely to become ill and have been noted to cause direct transmission of pneumonic plague [12-13]
Coxiella burnetii	Dogs, Cats	Contact with animal [12,14-15], environmental (dust inhalation) [14]	High concentrations of *C. burnetii* are present in animal birth products [12,14-15]
Bartonella henselae	Cats	Pet importation of infected flea [12,16], contact with animal (scratch > bite/lick) [12,16]	A rare cause of pulmonary disease with various manifestations [12]. Increased risk of bacillary angiomatosis in immunocompromised patients [12]
*Staphylococcus intermedius* group (SIG)	Dogs, Cats	Unknown	Only one case report of *S. intermedius *pneumonia was identified in the literature [3]. Other forms of SIG infection (cellulitis, bacteremia, upper respiratory tract) is known to be transmitted by saliva (bite/lick) or after an invasive procedure [17]
Multi-Drug Resistant Infections: Methicillin-Resistant *Staphylococcus aureus*, Methicillin-Resistant SIG, extended-spectrum β-lactamase producing Enterobacteriaceae	Dogs, Cats	Contact with animal [1-2,10], environmental contamination [1], nosocomial [1-2,10]	Pets may serve as a persistent reservoir for colonization or infection [1-2,10]

Bordetella (B.) bronchiseptica

There is often uncertainty behind the significance of finding *B. bronchiseptica *in cultures and its correlation with causing respiratory disease. The above case of *B. bronchiseptica* pneumonia with other respiratory co-isolates highlights the complicated course such infections may present. *B. bronchiseptica* is a gram-negative, aerobic coccobacillus that is peculiar in how it has been noted throughout the literature to accompany other pathogens in a polymicrobial fashion [4-5]. However, cases of *B. bronchiseptica *as the only identifiable source of infection have been identified [6]. B. bronchiseptica colonization is known to be a predisposing factor for secondary infections [4-5]. This increased propensity for infection is especially true for those with immunocompromising conditions. Cases of *B. bronchiseptica *have been documented to infect immunocompromised hosts, including those with human immunodeficiency virus (HIV), various hematologic malignancies, cystic fibrosis, rheumatoid arthritis, and lung cancer after chemotherapy [4-7]. One of the ways *B. bronchiseptica* is thought to predispose individuals to infection is by inhibiting leukocyte function via the production of adenylate cyclase toxin, which disrupts chemotaxis and superoxide production [4-5]. Unlike the closely related species *Bordetella pertussis*, *B. bronchiseptica* does not express the pertussis toxin, although it does possess the virulence gene within its DNA [6]. In addition, *B. bronchiseptica* can adhere to respiratory epithelial cells via fimbriae, pertactin, and filamentous hemagglutinin, which creates a difficult environment for mucus clearance [4-5].

The method of *B. bronchiseptica* transmission is still unclear. People who have close contact with their pets are at an increased risk for asymptomatic colonization or infection. The risk is further increased after contact with sick dogs or cats diagnosed with *B. bronchiseptic*a presenting as kennel cough [5-6]. Although vaccines exist for both cats and dogs against *B. bronchiseptica,* it is not considered a core vaccine and is often only administered to pets in high-risk situations such as being in a boarding facility or multi-pet household [18]. Immunization against *B. bronchiseptica* does not provide lifelong immunity and requires booster vaccination [18]. Other various mammal species can likewise acquire *B. bronchiseptica*, either as a colonizing organism or agent of disease. Interestingly, there are also case reports of nosocomial transmission to humans without traceable contact with animals [5]. It is unclear in the presented case if the patient had recent contact with animals. Unfortunately, upon transfer, she was in a precarious state with her relevant history provided by outside hospital documents and a healthcare surrogate. There is a possibility that her infection may have been nosocomial given that the tracheal aspirate cultures only grew MSSA while the BAL cultures obtained later were positive for *B. bronchiseptica* growth. Likely, there is more to the transmission of *B. bronchiseptica* than currently available data can provide.

The treatment of *B. bronchiseptica* can present a challenge. Antibiotic choices with adequate intracellular penetration are preferable as *B. bronchiseptica *can persist intracellularly and lead to chronic or recurrent infections. *B. bronchiseptica* is typically sensitive to tetracyclines, quinolones, aminoglycosides, antipseudomonal penicillins, and trimethoprim-sulfamethoxazole (TMP-SMZ) [5-6]. Rare cases of *B. bronchiseptica *resistance to TMP-SMZ have been reported [7]. This described case is noteworthy in that *B. bronchiseptica *demonstrated resistance to TMP-SMZ and only intermediate susceptibility to piperacillin/tazobactam, as outlined in Table 1. Macrolides are ineffective in treating *B. bronchiseptica*, which is often an empiric first choice for the treatment of community-acquired pneumonia [6]. Interestingly, antibiotic choices based on susceptibility patterns do not always correlate to clinical response. The clinical response to antibiotics can be complicated by the severity of the immunocompromising condition or other respiratory co-isolates [4-5]. Treatment duration can be as little as two to four weeks if a response is achieved or up to six months for recurrent or persistent infections [4-6]. The decision was made to view *B. bronchiseptica* in this case as asymptomatic colonization. If the treatment for each respiratory isolate had been pursued, the antibiotic choices would have been limited given the resistance patterns and the patient’s antibiotic allergies.

Pasteurella (P.) multocida

*P. multocida* is a gram-negative coccobacillus found in the respiratory passages of a multitude of mammals including cats and dogs. A history of exposure to dogs was verified in case two and contact with cats in case three. While *P. multocida *is typically associated with soft tissue infections, this pathogen is also implicated in severe respiratory infections, including pneumonia, lung abscesses, and empyema [8-9]. Transmission of respiratory infection from animal contact occurs after encountering saliva through bites or licking, scratches, and even inhalation [8-9]. A respiratory infection can develop either as a primary event after inhalation of droplets or acquired secondarily from bacteremic spread due to the various possible transmission methods [8].

Severe respiratory infections typically occur in patients who are elderly, immunocompromised, or have chronic pulmonary disease [8-9]. *P. multocida *can also cause systemic infection with bacteremia, more commonly in immunocompromised populations [8-10,19]. In case two, our patient had a pertinent history of COPD and untreated lung cancer. It is difficult to determine if *P. multocida* caused an active infection contributing to worsening respiratory symptoms or if the finding was more representative of colonization, given that her comorbidities could also explain her symptoms. In case three, BAL cultures did not grow *P. multocida*, but the bronchoscopy was performed days after the initiation of antibiotics for *P. multocida *bacteremia. This patient did not have any known prior lung disease; however, she did have a 15-pack-year smoking history as well as documented bronchiectasis on bronchoscopy.

Penicillin is considered the first-line treatment for *P. multocida *[8-9,19]. *P. multocida* is also commonly susceptible to amoxicillin-clavulanate, doxycycline, and fluoroquinolones [9-10]. Beta-lactamase production has been reported [9]. Macrolide use is not routinely recommended unless susceptibility is confirmed on testing due to higher macrolide resistance rates [19]. The patient in case two was empirically prescribed amoxicillin-clavulanate before final susceptibility testing resulted. The patient in case three was double-covered for *P. multocida *initially due to concern for possible resistance, given that she was recently treated with antibiotics that would be expected to cover this microbe adequately. In both cases, the isolates were susceptible to all tested antibacterial agents.

Francisella (F.) tularensis

Tularemia, a plague-like illness commonly known as ‘rabbit fever,’ is caused by the gram-negative coccobacillus called *F. tularensis*. Tularemia can have various presentations. Although the ulceroglandular form occurs most frequently, pneumonic forms of tularemia are present throughout the literature and have greater severity [11]. This pathogen has multiple transmission methods. The most common infection routes are through tick and deer fly bites or contact with infected animal carcasses, particularly rodents or lagomorphs [11]. However, cases of tularemia have been reported to be transmitted from cats and dogs, with cats being the more likely culprit [1,11]. Transmission routes from these pets include bites, scratches, and rarely inhalation [11-12]. Cats and dogs can also increase human exposure by importing infected ticks into the household [11]. Similar to *P. multocida*, a respiratory infection can develop as a primary event after inhalation or secondarily from bacteremia [11-12].

Yersinia (Y.) pestis

A rare cause of pneumonia comparable in many aspects to tularemia is *Y. pestis*, the gram-negative coccobacillus that causes plague. Similarly, *Y. pestis *presents with various forms of the disease. These forms include bubonic, pneumonic, and septicemic plague, with bubonic being the most common. In the absence of an epidemic, plague typically arises from the bite of an infected flea [13]. *Y. pestis* can also transmit via contact with an infected animal through bites, scratches, or inhalation [12-13]. While rodents are the first animals that come to mind regarding the spread of plague, both cats and dogs are potential agents for direct transmission. Cats are more likely to develop plague-related illnesses and consequently transmit the disease to humans compared to their dog counterparts [13]. Akin to tularemia, pneumonic plague arises as both a primary form of infection and secondarily from bacteremic spread [12-13].

Coxiella (C.) burnetii

*C. burnetii*, the agent of Q fever, is an interesting pathogen that has the potential to cause outbreaks of pneumonia. This microbe is an intracellular, gram-negative coccobacillus that can survive within the phagolysosomes of cells [14]. Transmission is most commonly thought to occur after inhalation of particles contaminated with infected bodily fluids from parturient ungulate species due to the propensity for *C. burnetii* to concentrate in the uterus of mammals [14-15]. Other mammals, including cats and dogs, have additionally been identified as sources of infection implicated in outbreaks of Q fever [1,12,14].* C. burnetii* can exist in various forms, including large cells, highly infectious small cells, and environmentally resistant spore-like particles [14]. Most mammals infected with *C. burnetii* are asymptomatic, aside from the increased risk of abortion [15]. Humans infected with *C. burnetii *often present with a self-limited febrile illness. However, 1-2% of acute infections ultimately develop pneumonia [14]. *C. burnetii* is also implicated in chronic disease which can manifest as interstitial lung fibrosis [14].

Bartonella (B.) henselae

*B. henselae* is not typically known as an agent for causing pneumonia. The most common presentation is the self-limited lymphadenitis called ‘cat scratch disease’ [12,16]. Another well-documented disease manifestation is bacillary angiomatosis, which presents as vascular nodules that can disseminate throughout the body in immunosuppressed patients. This gram-negative bacillus pathogen is still worth mentioning as there have been case reports of atypical cat scratch disease presenting with features of pneumonia and pleural effusions [12]. *B. henselae* has the ability to live intracellularly within erythrocytes, and transmission to humans is mainly through cat scratches [16]. There is also some evidence that transmission to humans can occur through cat saliva and possibly indirectly through contact with the cat flea, *Ctenocephalides felis* [12,16].

Multi-drug-resistant infections (MRSA)

Another important consideration of disease related to our companion animals is their potential as reservoirs to transmit resistant bacteria. MRSA is an acknowledged human pathogen recognized as an agent for a multitude of disease presentations, including pneumonia. Community-acquired strains of MRSA are growing in prevalence, with reports of transmission between humans and their pets, including cats and dogs [1,10]. One of the implications to consider is that a human treated for MRSA colonization or infection may ultimately be re-colonized or infected if MRSA colonization in the pet is unaddressed [10]. Similar implications have been noted for pets colonized with ESBL-producing Enterobacteriaceae [1-2].

Overall, *Staphylococcus (S.) aureus* is not the most predominant strain of staphylococcal species in cats and dogs. Rather, *S. intermedius* colonization is far more prevalent [10]. This gram-positive, coagulase-positive species also has the potential to develop methicillin resistance with evidence of carriage in humans and rare cases of infection [1,17]. Only one case of pneumonia due to *S. intermedius *has been reported in the literature in a patient five days post-coronary artery bypass grafting, interestingly with no known pet exposure [3]. Human infection with *S. intermedius* is likely more frequent than reported due to difficulties with identification [1,17]. Rapid coagulase tests are often falsely negative [17]. Even if coagulase is detected, *S. intermedius* may still be incorrectly identified as MRSA [17].

## Conclusions

This case series outlines current information on a selection of bacterial zoonoses that are potentially transmissible by cats and dogs with the ability to cause pneumonia. The clinical implication of this case series includes bringing to attention the impact that common household pets can play in the zoonotic transmission of respiratory illness. While these cases may not present frequently, they are still crucial to consider as part of the differential depending on the clinical scenario. A broadened differential is especially critical in immunocompromised individuals who may be at risk for rare pulmonary infections, including *B. bronchiseptica*, *P. multocida, and B. henselae*. One of the limitations of this case series is interpreting the significance of these microbes' impact on patients' illness courses. Each patient had other findings and complications related to their malignancies that can make it challenging to discern the role that infection played.

Other unusual scenarios, such as outbreaks of pneumonia caused by tularemia, plague, and Q fever, should be considered in the correct context. Although cats and dogs may not be the primary reservoirs for these diseases, their potential as a source of infection is not to be overlooked. This report also brings attention to the role that pets can play in harboring antibiotic resistance. With awareness comes the ability to attenuate risks for zoonotic transmission. Immunocompromised individuals can be advised to limit some types of close contact with pets, including bed-sharing and licking, especially in animals noted to be sick. Additionally, providers should counsel patients on the importance of proper flea control to prevent the spread of disease as well as regular surveillance for ticks that can be imported inside homes.
